# Evaluation of the Tempo® System: Improving the Microbiological Quality Monitoring of Human Milk

**DOI:** 10.3389/fped.2020.00494

**Published:** 2020-09-02

**Authors:** Marie-Pierre Cayer, Nathalie Dussault, Marie Joëlle de Grandmont, Marc Cloutier, Antoine Lewin, Danny Brouard

**Affiliations:** ^1^Affaires Médicales et Innovation, Héma-Québec, Québec, QC, Canada; ^2^Département de biochimie, Université Laval, Québec, QC, Canada; ^3^Département d'obstétrique et gynécologie, Université de Sherbrooke, Québec, QC, Canada; ^4^Département de chimie, Université Laval, Québec, QC, Canada

**Keywords:** donor human milk, human milk bank, bacteriology testing, donor screening, *Bacillus*, quality control, in-process monitoring

## Abstract

**Background:** Bacteriological testing of donor human milk is mostly done both before and after pasteurization to control contamination in the end-product and meet the microbiological standards. Although the plate count method represents a reliable and sensitive technique and is considered the gold standard for bacteriological testing, it is recognized for being time-consuming and requiring qualified personnel. Recently, faster testing technologies, mostly geared toward the food industry, have been developed. Among these, the bioMérieux TEMPO® system uses the most probable number method to assess microbiological content in a semi-automated fashion.

**Objective:** The performances of the TEMPO® system in enumerating bacterial quality indicators in human milk were assessed and compared to the reference plate count method.

**Methods:** Naturally and artificially contaminated human milk samples were used to compare the analytical performances of the TEMPO® system to the plate count technique. More specifically, bacteria belonging to the genera *Bacillus, Enterobacteriaceae, Staphylococcus aureus*, and total aerobic flora were screened using both methods. Bacteria isolated on agar plates containing selective media were identified by supplemental testing. Bacterial testing results and method parameters were compared using linear regression analyses and Bland-Altman approaches.

**Results:** Naturally contaminated milk samples (*n* = 55) tested for total aerobic flora showed < 1 log (CFU/ml) discrepancy between the two methods in the output results for 98% of the samples. Comparative linear regression analyses demonstrate good correlations between the two methods (*R*^2^ > 0.9). At lower levels of bacterial contamination, the TEMPO® method precision (C.V. < 8%) and accuracy (> 83%) were comparable to plate counts.

**Conclusions:** The analytical performances of the TEMPO® system for human milk bacteriological testing are equivalent to the reference plate count method. Results from the TEMPO® system are available within a 24-h turnaround time from sample inoculation without the need for further supplemental testing, suggesting that this semi-automated method could be implemented within milk bank operations as an in-process monitoring technology to optimize end-product quality and safety.

## Introduction

A human milk diet has multiple, well-established benefits for all infants and reduces many risks associated with prematurity. Human milk banks provide an essential source to allow human milk diet for infants whose needs exceed what their own mothers can provide. These institutions need to follow standard safety guidelines regarding end-product bacteriological screening to ensure product safety (1–3). Since the inception of its Public Mother's Milk Bank in 2014, Héma-Québec has overseen the milk donation process and is responsible for the processing and distribution of pasteurized donor human milk (PDHM) to premature infants born at 32 weeks or earlier who require medical care and whose mother is unable to breastfeed. Recent studies have shown that breast tissue itself may contain several hundred bacterial species whose diversity may vary according to lactation stages (4–6). Although a normal milk microflora contributes to the establishment of a healthy intestinal flora in neonates, harmful pathogens in human milk can resist thermal processing and have been related to premature babies' infections (7–11).

Bacterial content analysis of pooled mother's milk prior to thermal processing helps identifying potential sources of contamination and allows educational feedback to donors when inadequate collection processes are suspected. The Human Milk Banking Association of North America (HMBANA) guidelines provide recommendations on bacteriological testing of heat-processed milk. Specifically, the guidelines states that any bacterial growth is deemed unacceptable for banked human milk (1). These recommendations on PDHM have been implemented by most milk banks. However, bacteriological screening of milk before pasteurization has not been systematically implemented in all milk banks and there is still no consensus regarding the microbiological criteria to be applied (12–14). Monitoring contaminants in “raw” milk remains relevant, since some pathogens produce toxins or spores that may resist thermal processing (15, 16). Spore-forming bacteria including *Bacillus* and *Bacillus*-derived bacterial genera species, represent the most common contaminants found in PDHM and are responsible for a significant fraction of rejected batches (14, 17). Most milk banks who apply pre-pasteurization milk screening use a total aerobic flora (TAF) threshold of 10^5^-10^6^ colony-forming units per milliliter (CFU/ml) (18, 19). Threshold values for maximal bacterial counts vary among milk banks and countries. For instance, the National Institute for Health and Care Excellence (NICE) in the UK recommends a maximal bacterial count of 10^4^ CFU/ml for *Staphylococcus aureus* (*S. aureus*) and *Enterobacteriaceae*, and maximum of 10^5^ for total colony count. In contrast to Australian guidelines, which do not accept any *Enterobacteriaceae*, Enterococci or potential pathogens capable of producing heat-stable enterotoxins (2, 20–22). Héma-Québec's Public Mother's Milk Bank adopted post-pasteurization microbiological criteria based on practices and guidelines; accordingly, unpasteurized milk containing a TAF > 10^5^ CFU/ml or an *Enterobacteriaceae* content > 10^4^ CFU/ml or any harmful pathogens including *S. aureus, Bacillus*, and *Bacillus*-like species, is discarded.

The plate count method (PCM) is currently the best practice recommended by the HMBANA for bacterial content testing of milk (1). This method can require up to 4 days for the results to be available and its application might lack standardization since procedure variations such as media composition, sample plating volume and raw milk preparations (i.e. dilutions) have been observed among milk banks (23–25). This methodology precludes a rapid intervention upon the human milk processing chain and can result in time, material and product wastage.

New bacteriological testing technologies with higher throughput have been recently introduced, among others, by the food and cosmetic industries as a means of intervening faster on their production chain (26, 27). The TEMPO® system (bioMérieux, Marcy-l'Étoile, France) is the first semi-automated bacteriological testing technology able to identify contaminants typically found in human milk. This system uses testing cards with specific culture media allowing rapid bacterial growth. After distribution of culture media-sample mixtures in the wells of the card, bacteria multiply during incubation and metabolize culture media containing a fluorescent indicator. The TEMPO® system relies upon the detection of the number and volume of positive wells (fluorescent or non-fluorescent) and statistical methods that are based on the most probable number (MPN) approach to perform bacterial enumeration (28, 29). The TEMPO® system is increasingly used in the food industry, which takes advantage of its semi-automated process, built-in sample scanner for traceability purposes and its speed in achieving bacteriological count and identification without additional identification tests (30, 31). Human milk and dairy products have similar matrices in terms of fat and inhibitory substances, which suggest that the TEMPO® system might be an asset for milk banks. This technology could potentially be used as a faster means to perform in-process bacteriological monitoring of raw mother's milk and PDHM (32, 33).

The objective of this study was to conduct a performance evaluation of the TEMPO® system for the enumeration of four typical bacterial quality indicators observed in mother's milk (TAF, *Enterobacteriaceae, S. aureus*, and *Bacillus*) and to compare the results with standard PCM. The impacts of the milk matrix on the reliability of the TEMPO® method were assessed along with the precision and accuracy. To the best of our knowledge, this study represents the first assessment of the TEMPO® system performances for human milk bacterial testing.

## Materials and Methods

### Milk Samples

Before collection, donors were asked to fill a milk donation qualification form providing relevant personal and medical information and to sign an informed donation consent form, in accordance with Héma-Québec's (HQ) Research Ethics Committee guidelines. Qualified donors were subjected to the same serological screening performed for regular blood donations at HQ's blood bank (i.e., hepatitis B and C, syphilis, HIV, CMV, and HTLV-I/II). Expressed milk was self-collected and frozen in a household refrigerator by mothers at their home. Periodically, milk was sent to the Public Mothers' Milk Bank using validated HQ shipping containers. All milk donations were stored at −20°C until analysis. On the day of the analysis, milk samples were rapidly thawed in a water bath at 37°C. Naturally Contaminated Milk Samples (NCMS), and uncontaminated milk samples sterilized by pasteurization were spiked with known concentrations of specific bacterial strains and labeled as Artificially Contaminated Milk Samples (ACMS). *Escherichia coli* (ATCC 25922), *Staphylococcus aureus* (ATCC 27217) and *Bacillus cereus* spores (isolated from a milk donation) were used to prepare ACMS. Mother's milk bacteriological contents were confirmed by PCM and diluted to final target concentrations in order to generate ACMS. For NCMS (*n* = 55) and ACMS (*n* = 3), 10-fold serial dilutions (10^−1^, 10^−2^, or 10^−3^) were prepared in Buffered Peptone Water (BPW; bioMérieux) for bacterial enumeration, as described in Table 1.

**Table 1:** Protocol details for human milk bacterial testing.

	**Total aerobic flora**		***Enterobacteriaceae***		***S. aureus***		***Bacillus cereus*****group**	
**Conditions**	**TEMPO^®^**	**PCM**	**TEMPO^®^**	**PCM**	**TEMPO^®^**	**PCM**	**TEMPO^®^**	**PCM**
Initial sample dilutions	10^0^, 10^2^	10^0^-10^3^	10^1^	10^0^-10^3^	10^0^	10^0^	10^0^	10^0^-10^3^
Sample volume (mL)	1	0.1	1	0.1	1	0.1	1	0.1
Agar media or TEMPO® tests	AC	Blood sheep	EB	MacConkey	STA	Mannitol-salt	BC	Blood sheep
Incubation period (h)	24	48	22	24–48	24	48	22	24–48
Incubation temperature (°C)	35	35	35	35	35	35	30	30
Detection method	Fluorescence+	All colonies	Fluorescence–	Purple colonies	Fluorescence–	Yellow colonies	Fluorescence+	Hemolysis
Supplemental tests	No	No	No	TSI^*^	No	Coagulase	No	Sporulation

* *Triple-sugar iron agar*.

### Enumeration of Bacterial Quality Indicators by the Plate Count Method (PCM)

The PCM, performed by the spread plate technique, was used to enumerate all four bacterial quality indicators in milk samples. The method's performances had been previously validated following controlled quality assurance protocols. Briefly, 100 μl of milk sample were seeded on different media in duplicate. Plates were inverted and incubated at 35°C for 48 h. For the enumeration of TAF and *Bacillus* species, sheep blood agar medium was used (Oxoid; Thermo Fisher Scientific, Waltham, MA, USA). Colonies characterized by a clear zone halo, which can be attributed to hemolytic activity, were ascribed to *Bacillus*. After isolation on blood sheep agar, gram staining and inoculation of sporulation agar medium were performed to confirm the *Bacillus* identification. *Enterobacteriaceae* and *S. aureus* were enumerated on MacConkey and mannitol medium (Laboratoire de santé publique du Québec, Québec), respectively. For NCMS, characterized by a competitive flora, only presumptive pink to purple colonies fermenting lactose were counted as *Enterobacteriaceae*. Triple-Sugar-Iron agar (TSI) medium plates were seeded with bacteria to confirm the presence of *Enterobacteriaceae*. *S. aureus* in NCMS could not be quantified using the previous PCM because coagulase-negative *Staphylococcus* (CONS) and coagulase-positive Staphylococci (CPS; *S. aureus*) could both grow in mannitol medium. CPS identification was confirmed by the observation of presumptive bright yellow colonies on sheep blood agar after a 24-h incubation period at 35°C. CPS was differentiated from CONS using a coagulase test, following the manufacturer's instructions (BD BBL™ Rabbit Coagulase Plasma). For all samples, average counts from duplicates are reported as original concentrations, considering the applied dilution factor.

### Enumeration of Bacterial Contaminants by the TEMPO® MPN Method

The bioMérieux TEMPO® system consists of two independent work stations, the semi-automated sample preparation module and the reading/recording module. In this study, the TEMPO® system was used according to the manufacturer's recommendations. Briefly, each individual bacterial analysis was performed using its own specific testing card, which contains a growth-triggered fluorescent transducer scattered in a selective culture medium and a predefined sample dilution pattern for determination of the MPN. Each card contains 48 analysis wells of three different volumes (225, 22.5, and 2.25 μl), from which a fluorescence signal can be recorded and interpreted to deduce the initial bacterial content reported in CFU/ml.

Before each bacterial analysis, a 1 mL milk sample was transferred into a previously rehydrated disposable vial containing 3 mL of sterile water. The seeded medium was subsequently transferred to its attached testing card and sealed using the TEMPO® Filler. Each testing card was incubated for a specific time and temperature, following the manufacturer's recommendations (see Table 1). In this study, the TEMPO® AC testing card was used to characterize TAF and was incubated for 24–28 h at 35°C before reading. *Enterobacteriaceae* were enumerated using the TEMPO® EB testing card and was incubated at 35°C for 22–27 h. The TEMPO® STA testing card was used to determine the *S. aureus* concentration at 35°C for 24–27 h. Finally, a 22–27 h incubation period at 30°C was used with the TEMPO® BC testing card to assay *Bacillus* species belonging to the *Bacillus cereus* group (i.e., *B. cereus, B. anthracis, B. thuringiensis, B. mycoides, B. pseudomycoides, B. weihenstephanensis*, and *B. cytotoxicus*). After their respective incubation period, all cards were analyzed sequentially in an automated fashion using the TEMPO® reader station. Results are reported in CFU/ml for the original sample after manually entering the initial dilution factor (34).

### Comparison of the Methods' Performances TEMPO® vs. PCM

A total of 55 NCMS were tested for TAF and *Enterobacteriaceae* by the TEMPO® MPN and PCM methods, and results were directly compared. Since the TEMPO® BC testing card is specific to the *Bacillus cereus* group, comparison of enumeration results with PCM, which uses blood sheep agar allowing growth of other *Bacillus* species, could not be done. Since CONS and CPS could both grow in mannitol medium, the measured concentration of *S. aureus* could also not be determined by PCM. Hence, the comparison of methods' performances for *S. aureus* and *Bacillus* detection was made from the number of positive samples (% of the population of *n* = 55) analyzed by both methods. Regarding the PCM method for TAF, *Enterobacteriaceae* and *Bacillus*, plate counts were obtained by seeding the undiluted sample along with 10^−1^, 10^−2^, and 10^−3^ dilutions. Detection of *S. aureus* was performed by spreading the original undiluted milk sample. Every plate was prepared in duplicate, and results are reported as the average count from both plates.

For TEMPO® measurements, two TEMPO® AC testing cards seeded, respectively, with 10^0^ and 10^−2^ dilutions of the undiluted milk sample were used to cover the TAF enumeration range (1-490 000 CFU/ml, or 0–5.7 log CFU/ml). TEMPO® EB testing cards were seeded with 10^−1^ dilutions giving a detection range of 10–49 000 CFU/ml, or 0–4.7 log CFU/ml. Finally, the TEMPO® STA and BC testing cards were also seeded with the original milk sample (i.e., 10^0^ dilution), and their enumeration range is 1–4,900 CFU/ml, or 0–3.7 log CFU/ml.

### Evaluation of the Analytical TEMPO® MPN Method Parameters

The accuracy of the TEMPO® method for each bacterial quality indicator and the milk matrix effect were assessed using the corresponding PCM results as reference. A high concentration of NCMS was used for the determination of the TEMPO® accuracy and precision for the enumeration of TAF (i.e. 5 log CFU/ml). ACMS were prepared by spiking *Escherichia coli* for *Enterobacteriaceae* enumeration, *Bacillus cereus* spores for *Bacillus* testing and *S. aureus* for the detection of CPS. PDHM or BPW was used as diluent to decrease the bacterial concentration. To cover an enumeration ranging from 1 to 5 log CFU/ml, 10^0^-10^−2^ sample dilutions were seeded on plates or TEMPO® cards.

#### Selectivity and Linearity

To assess the impacts of the milk matrix on the reliability of the TEMPO® method, milk samples were diluted in PDHM or BPW to obtain standard concentrations ranging between 1 and 5 log CFU/ml for TAF and *Enterobacteriaceae*, and between 1 and 4 log CFU/ml for *S. aureus* and *Bacillus cereus*. From each standard, four 10-fold serial dilutions were prepared for TAF, and five 10-fold serial dilutions were needed for *Enterobacteriaceae, S. aureus* and *Bacillus cereus* enumeration tests. Negative control samples previously determined to contain 0 CFU/ml were also prepared and analyzed. The TEMPO® and PCM enumeration results for each bacterial quality indicators diluted in either pasteurized milk or BPW were compared by regression analysis. All experiments were performed in triplicate.

#### Accuracy and Precision

The precision of each method was assessed from six individual measurements of six test samples prepared from the same mother's milk sample. The accuracy of the analytical method for each bacterial quality indicator was determined using three individual test samples prepared from mother's milk samples, and final target concentrations were 5 log CFU/ml for TAF, 4 log CFU/ml for *Enterobacteriaceae*, 3 log CFU/ml for *S. aureus* and 1 log CFU/ml for *Bacillus cereus*. All test samples were analyzed using both the PCM and TEMPO® method. Seeding on media or TEMPO® cards was performed using sample dilutions of 10^−2^ for TAF, 10^−1^ for *Enterobactericeae* and *S. aureus*, and no dilution (10^0^) for *Bacillus cereus*. The results of the precision tests are expressed by the coefficient of variation (CV), which is the relative standard error compared to the mean. The method's accuracy (%) for each quality indicator was calculated by reporting the experimentally measured concentrations to the expected values.

### Statistical Analysis

Bacterial counts were converted to logarithmic values. The latter were analyzed with standard statistical methods for means and standard deviations (SD). The correlation between the PCM and TEMPO® results was assessed by linear regression analysis and the coefficient of determination (*R*^2^) was used to estimate the model accuracy. The agreement between methods was evaluated by performing a Bland-Altman analysis (OriginPro8.0, OriginLab Corporation, Northampton, MA, USA; SAS 9.4, SAS Institute, Cary, NC, USA).

## Results

### Comparison of TEMPO® and PCM Performances With NCMS

Samples (*n* = 55) were tested for all four bacterial quality indicators by both methods. Comparing bacterial counts obtained for each sample tested by the PCM and TEMPO® methods, there was no more than a 1 log CFU/ml difference in the results, irrespective of the bacterial target.

#### Total Aerobic Flora

**Figure 1:**
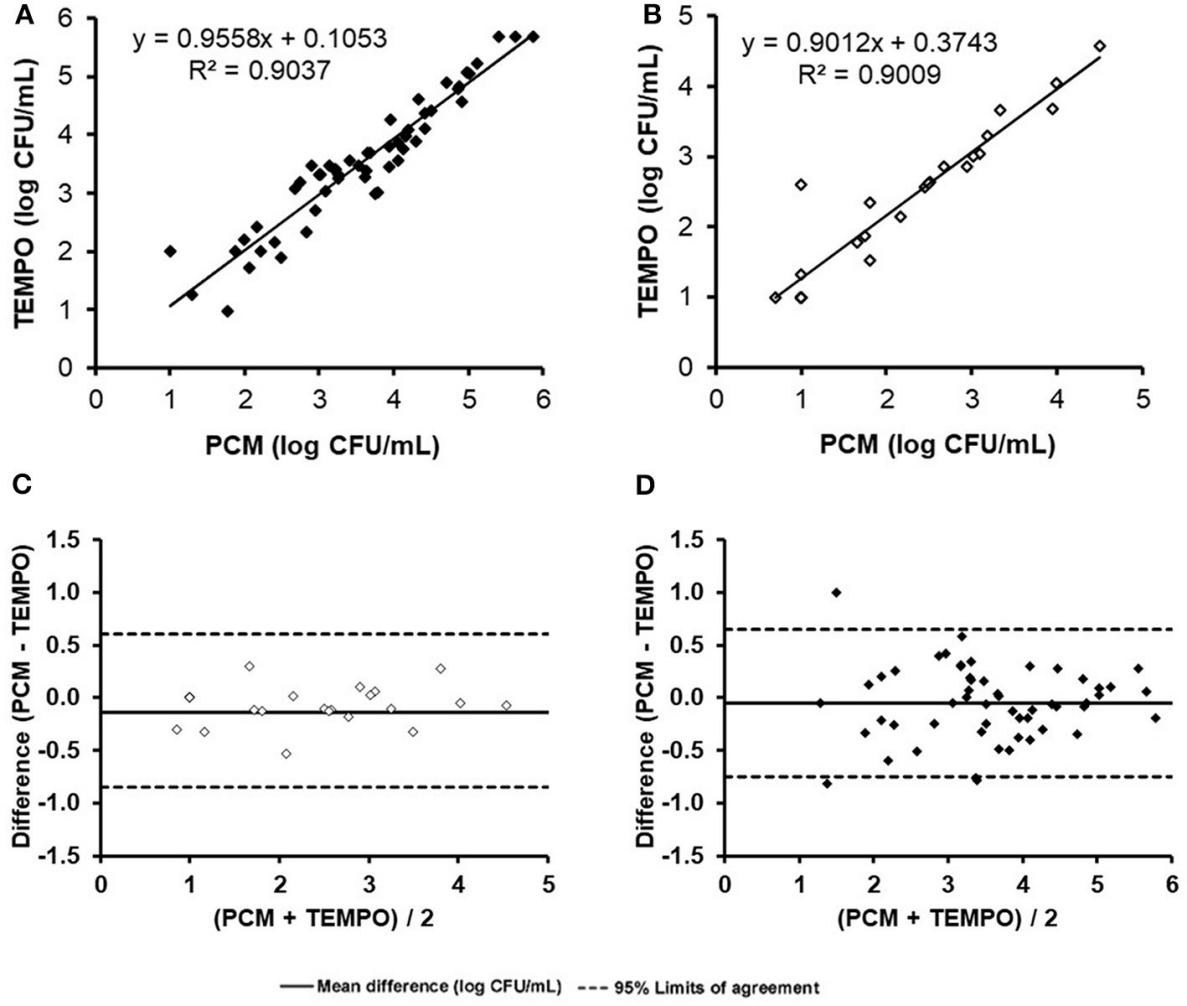
Comparison of total aerobic flora and *Enterobacteriaceae* enumerations (log count per mL) for the 55 naturally contaminated samples by the TEMPO® and plate count methods. Linear regression curve **(A)** and Bland-Altman analysis **(C)** for total aerobic flora. Linear regression curve **(B)** and Bland-Altman analysis **(D)**.

When comparing enumeration results obtained from both methods for TAF using a linear regression analysis, the coefficient of determination obtained was 0.90 (Figure 1A). The concentration of one milk sample was out of the quantification range for both methods, and was therefore excluded from the analysis. According to the Bland-Altman analysis (Figure 1C), there was a good agreement between both methods, with a mean difference of < 0.5 log CFU/ml across the entire range of values. Only a few samples (*n* = 4) were found to be outside the agreement limits of the Bland-Altman analysis set at 2 × SD. The degree of correlation and agreement between measurements is highlighted by the narrow 95% CI (−0.8 to 0.7 log CFU/ml) and an analytical bias of only 0.1 log CFU/ml for the TEMPO® method over the PCM.

#### Enterobacteriaceae

Only 44% (24/55) of milk samples were found positive for *Enterobacteriaceae* after analysis by both methods. At low concentrations (< 1.3 log CFU/ml), two samples were positive according to the TEMPO® EB test, but only one showed growth by PCM using MacConkey agar medium (Table 2). A statistical analysis was performed on the 24 positive samples, and a linear regression coefficient of 0.90 was obtained (Figure 1B). The Bland-Altman plot (Figure 1D) shows a good agreement between the two methods, as all results are within the 95% C.I. limits (−0.9 to 0.6 log CFU/ml), with an analytical bias of only 0.1 log CFU/ml and a calculated ICC of 0.94, suggesting an excellent reliability for the TEMPO® method.

**Table 2:** Comparative detection of four bacterial quality indicators in 55 NCMS using TEMPO® and PCM methods.

		**PCM**							
		**Total aerobic flora**		***Enterobacteriaceae***		***S. aureus***		***Bacillus sp***.	
		**Positive**	**Negative**	**Positive**	**Negative**	**Positive**	**Negative**	**Positive**	**Negative**
**TEMPO method**	**Positive**	55	0	24	2	1	0	4	5
	**Negative**	0	0	1	28	2	52	1	45

#### S. aureus

*S. aureus* was detected by the PCM and the strain identity was confirmed by the coagulase test (Table 2) for three of the 55 milk samples (5.4%). The TEMPO® STA test gave a positive result for only one of them associated with an error (code 19). The code 19 is associated to an inconsistent result generated by the TEMPO® reader system. When an incoherent result is obtained, fluorescence is present in the upper wells of the card, confirming the presence of bacteria in the sample, but without a concentration estimate. Despite the fact that it was not possible to obtain a bacterial count for this sample due to a code 19, but considering that the error is associated with growth, they were identified as being contaminated. Consequently, there were two human milk samples unconfirmed positive for the presence of *S. aureus* by the TEMPO® system out of the 55 analyzed.

#### Bacillus

*Bacillus* was detected in nine of the 55 samples by TEMPO®, compared to only five by the reference PCM. Only one of the five positive samples identified by PCM was detected exclusively by the gold standard method. On the other hand, five of the nine positive samples by TEMPO were considered negative by PCM. In the end, four samples were tested positive for *bacillus* by both methods (Table 2). Interestingly, the single colony of a presumptive *bacillus* that grew on blood agar did not generate beta hemolysis, thereby questioning the identity as a *cereus* species. However, analysis by mass spectrometry (MALDI-TOF) confirmed that the bacterium belongs to the cereus group, and therefore should have been detected by the TEMPO® BC test; however the concentration was only 5 CFU/ml. Overall, both methods demonstrated their capacities to detect low levels of *bacillus* i.e., from 0 to 3.7 log CFU/ml (1–4,900 CFU/ml).

### Evaluation of the Analytical TEMPO® MPN Method Parameters

#### Selectivity and Linearity

The TEMPO® system enables the quantification of bacterial quality indicators over a concentration range of four to five orders of magnitude. To assess the impacts of the complex milk matrix on the TEMPO® method's analytical performances, enumeration results for all bacterial target were compared in ACMS vs. BPW, with the exception of TAF, which was performed in NCMS only (Table 3). The statistical analysis shows a good overall agreement between the PCM and TEMPO® methods for all tested bacterial strains and sample matrices. More specifically, the linearity coefficient between the PCM and TEMPO® results for the enumeration of the TAF in milk is *R*^2^ = 0.976. Furthermore, bacterial counts obtained for *Enterobacteriaceae* by the PCM using MacConkey medium correlated just as well in a matrix composed of either milk (*R*^2^ = 0.995) or BPW (*R*^2^ = 0.996). The linear correlation coefficients observed when plotting *S. aureus* counting results by TEMPO® vs. PCM are *R*^2^ = 0.990 and *R*^2^ = 0.948 for BPW and milk matrices, respectively. Finally, there were no significant differences in the results obtained by the two methods for the enumeration of *B. cereus* using BPW (*R*^2^ = 0.994) or milk (*R*^2^ = 0.990) samples artificially contaminated with spores. Note that an *R*^2^ value of 1.0 indicates a perfect fit between variables and indicating that the model explains all the relationship, between the two factors.

**Table 3:** Correlation coefficients between plate count and TEMPO® methods for the enumeration of bacterial quality indicators in ACMS and NCMS contaminated samples.

	**Total aerobic**	***Enterobacteriaceae***	***S. aureus***	***Bacillus***
	**flora**			***cereus***
Enumeration range^*^ (log/ml)	0–5	0–5	0–4	0–4
Correlation (*R*^2^)				
NCMS	0.976	n.a.	n.a.	n.a.
ACMS buffered peptone water	n.a.	0.996	0.990	0.994
Artificially contaminated milk	n.a.	0.995	0.948	0.990

* *A minimum of four dilutions were teste (n = 3)*.

*n.a., not available*.

#### Precision and Accuracy

Table 4 presents the results obtained for the characterization of the precision and accuracy of both methods. For the precision expressed as the CV, a series of six measurements were taken from samples prepared so that their final concentration was strategically located within the quantification range of each bacterial target. For TAF, the expected sample concentration was 5 log CFU/ml. A CV of 3% was obtained for TEMPO, compared to 2% for PCM for paired samples. For *Enterobacteriaceae*, the enumeration target was set to 4 log CFU/ml and, similar to the TAF result, a slightly lower CV was obtained for PCM (1%) compared to TEMPO (3%), which was identical to the CV for TAF. A lower target concentration of 3 log CFU/ml was chosen for *S. aureus*, given the more stringent human milk release criteria for gram-positive bacteria. For this target species and concentration, CV of 5 and 2% were obtained for TEMPO® and PCM, respectively. A low 1 log CFU/ml concentration target was chosen for *Bacillus*; given this target, a CV of 9% was obtained with TEMPO®, and a much higher variability was observed in the results for PCM, with a CV of 82%.

**Table 4:** Evaluation of the accuracy and precision of the plate count and TEMPO® methods at concentrations close to the acceptance criteria for human milk before pasteurization.

	**Expected**	**Measured**	**CV (%)**	**Accuracy^**¥**^**
	**concentration**	**concentration**		**(%)**
	**(log CFU/ml)**	**(log CFU/ml)**		
**Plate count method**				
Total aerobic flora	5.3	5.2 ± 0.1	1.7	98.1
*Enterobacteriaceae*	4.0	4.0 ± 0.1	1.4	100.0
*S. aureus*	3.0	2.7 ± 0.1	2.0	90.0
*Bacillus cereus* (spores)	1.0	0.8 ± 0.6	81.6	80.0
**TEMPO**^®^ **method**				
Total aerobic flora	5.3	5.3 ± 0.1	2.9	100.0
*Enterobacteriaceae*	4.0	4.1 ± 0.1	3.0	102.5
*S. aureus*	3.0	2.5 ± 0.1	5.3	83.3
*Bacillus cereus* (spores)	1.0	1.2 ± 0.1	9.1	120.0

¥ *Accuracy = (measured concentration/expected concentration) ×100 (n = 3)*.

*Coefficient of variation = (standard deviation/mean) ×100 (n = 6)*.

The accuracy of each method was calculated from the difference observed between the average experimental value, calculated from three separate measurements, and the expected value for each sample whose final concentration was adjusted so as to be in the quantification range. Table 4 presents the PCM and TEMPO® accuracy results observed for each target bacterium. In the case of TAF and *Enterobacteriaceae*, values approaching 100% were obtained with both enumeration methods at 4 and 5 log CFU/ml, respectively. For *S. aureus*, the target concentration to assess the accuracy of the PCM and TEMPO® methods was 3 log CFU/ml; at that concentration, the accuracy was 90 and 83% for PCM and TEMPO® method, respectively. Finally, a 1 log CFU/ml target concentration was used with *Bacillus*; at that target, the PCM method underestimated the milk sample contamination (80%), and the TEMPO® method overestimated it (120%).

## Discussion

The TEMPO® system is currently used in several countries, mainly as a quality control tool in the food industry. Indeed, all TEMPO® bacterial tests have been approved by international quality and standards organizations, such as AOAC International, AFNOR and ISO for food and environmental samples (35–38). Evaluations of the performances of the TEMPO® MPN method for the characterization of bacterial content in food products, including dairy milk, have been reported (39–45). Most of them have demonstrated a good agreement in the results between the plate count method and TEMPO® for all types of targeted bacteria. The main objective of this performance evaluation was to compare the enumeration capability of the TEMPO® system aimed at the characterization of human milk microflora and the detection of specific bacterial contaminants of interest at concentration levels as low as 1 log CFU/ml in < 24 h. The enumeration results for TAF and *Enterobacteriaceae* obtained from 55 NCMS demonstrated a good agreement between the PCM and TEMPO® methods and, more importantly, no significant analytical bias was observed for the TEMPO® system using plate counts as reference. Differences in the output values were not > 1 log CFU/ml for all samples, which testify to the reliability of the TEMPO® system. Interestingly, occasional discrepancies were observed between enumeration counts obtained from TEMPO® AC cards inoculated with 10^0^ and 10^−2^ dilutions of the same mother's milk sample. The upper detection limit threshold of 490 000 CFU/ml was exceeded with the undiluted sample, while the 10^−2^ dilution generated bacterial counts within the 1–4,900 CFU/ml enumeration range. This result could be attributed to an apparent fluorescence quenching caused by diffusion or absorption of the output signal by the complex milk matrix, interpreted by the system as a sample of high bacterial content. A dilution of the sample seems to attenuate the matrix effect on the fluorescence output signal and allows obtaining results within the quantification range of the TEMPO® AC card. This in turn enables to identify milk samples with an aerobic flora content that exceeds the quality criterion of > 10^5^ CFU/ml with a good level of confidence.

Testing a 10^−1^ sample dilution would be recommended for the enumeration of *S. aureus*; this dilution, corresponding to a limit of detection of < 10 CFU/ml, conforms to the quality criterion threshold for this bacterial species and corresponds to the current plate count method performed on mannitol agar in terms of limit of detection. Finally, 10^0^ and 10^−1^ dilutions of the mother's milk sample would be recommended to span appropriate concentration ranges for the enumeration of *Bacillus* and *Enterobacteriaceae* with TEMPO® BC and EB cards, according to the quality criteria applicable to these bacterial species. During the evaluation of the TEMPO® analytical performances, a few samples, independently of the targeted bacteria, generated an error code (code 19), which could be attributed to incoherent results according to the TEMPO® user's manual. The error code is believed to be associated to the opacity of the human milk matrix since all events were observed with undiluted samples. Light absorption by proteins or scattering by the milk fat globules, among others, could interfere with the bacterial growth transducer excitation of its fluorescence emission resulting in a noisy optical signal. According to bioMérieux's technical staff, adherence to the incubation period recommended for each testing card should allow sufficient growth from the lower wells and solve the issue. Repeating the same sample analyses with new testing cards and using the upper recommended limit as the incubation period, the same error code was obtained. One must conclude on the possibility that some human milk constituents may interfere with the TEMPO® detection process. Proper identification of the principal interfering constituents and, extensively, of maternal factors impacting detection performances of the TEMPO® system could be investigated. Nevertheless, to maintain a detection limit of 1 CFU/ml for *S. aureus* and *Bacillus* sp. it requires performing TEMPO® analyses on undiluted samples. Consequently, it would be well-advised to conduct secondary testing in the case of positive samples for *S. aureus* or *Bacillus* sp. in order to determine the bacterial concentration. It is worth noting that some *Bacillus* species outside the *cereus* group would not be detected by the TEMPO® BC test. However, this test remains efficient at quantifying some of the most harmful contaminants with pathogenic potential related to this genus (46). Finally, TEMPO®'s precision and accuracy were comparable to the PCM and, interestingly, the semi-automated detection method performed better than the PCM at low concentration levels, which might prevent the distribution use of human milk contaminated with small numbers of bacteria belonging to the *Bacillus cereus* group.

Within a 24-h period, characterization of the bacterial content in mother's milk donations and pasteurized human milk bottles were performed using the TEMPO® technology from bioMérieux. More specifically, TEMPO® was used for the enumeration of the total aerobic bacterial flora, *Enterobacteriaceae, B. cereus* group and *S. aureus* from raw human milk with sample dilutions as the only sample preparation steps prior to the incubation and detection. Rapid and reliable detection of bacterial content would help screening for contaminated milk donations with heat-resistant bacteria prior to pasteurization, thereby reducing rejection rates, and improving overall end-product safety and quality. The bioMérieux TEMPO® system includes a built-in scanning device ensuring sample traceability. It is designed to be integrated within a human milk production line for in-process testing and determining pooling strategies before thermal processing. This study underlines the importance for human milk banks to keep an eye on innovative technologies, especially those dealing with the characterization of bacterial content, as these could lead to enhancements in productivity but most of all, in the safety of a precious product intended for preterm infants.

## Data Availability Statement

All data sets are stored on a dedicated external hard drive. They can be made available upon request to the corresponding author.

## Ethics Statement

The studies involving human participants were reviewed and approved by the legal affairs department at Héma-Québec. The patients/participants provided written informed consent to participate in this study.

## Author Contributions

M-PC, MG, and DB conceived and designed the study and wrote the article. ND collected the data and contributed to the discussion of the results. AL performed the statistical analysis. MC provided suggestions concerning the content of the article and were responsible for critically revising the manuscript. All authors contributed to the article and approved the submitted version.

## Conflict of Interest

The authors declare that the research was conducted in the absence of any commercial or financial relationships that could be construed as a potential conflict of interest.
